# Fibrosis of Peritoneal Membrane as Target of New Therapies in Peritoneal Dialysis

**DOI:** 10.3390/ijms23094831

**Published:** 2022-04-27

**Authors:** Valentina Masola, Mario Bonomini, Silvio Borrelli, Lorenzo Di Liberato, Luigi Vecchi, Maurizio Onisto, Giovanni Gambaro, Roberto Palumbo, Arduino Arduini

**Affiliations:** 1 Department of Biomedical Sciences, University of Padova, 35131 Padova, Italy; maurizio.onisto@unipd.it; 2 Department of Medicine, Section of Nephrology and Dialysis, G. d’Annunzio University, 66013 Chieti, Italy; mario.bonomini@unich.it (M.B.); lorenzo.diliberato@asl2abruzzo.it (L.D.L.); 3 Unit of Nephrology, Department of Advanced Medical and Surgical Sciences, University of Campania “Luigi Vanvitelli”, Piazza Miraglia, 80138 Naples, Italy; dott.silvioborrelli@gmail.com; 4 Unit of Nephrology, Santa Maria Hospital, 05100 Terni, Italy; pvvecchi@gmail.com; 5 Division of Nephrology and Dialysis, Department of Medicine, Piazzale A. Stefani 1, 37126 Verona, Italy; giovanni.gambaro@univr.it; 6 Unit of Nephrology, Sant’Eugenio Hospital, 00144 Rome, Italy; palumbo.dr@gmail.com; 7 R&D Department, Iperboreal Pharma, 65122 Pescara, Italy; a.arduini@iperboreal.com

**Keywords:** peritoneal dialysis, biocompatibility, fibrosis

## Abstract

Peritoneal dialysis (PD) is an efficient renal replacement therapy for patients with end-stage renal disease. Even if it ensures an outcome equivalent to hemodialysis and a better quality of life, in the long-term, PD is associated with the development of peritoneal fibrosis and the consequents patient morbidity and PD technique failure. This unfavorable effect is mostly due to the bio-incompatibility of PD solution (mainly based on high glucose concentration). In the present review, we described the mechanisms and the signaling pathway that governs peritoneal fibrosis, epithelial to mesenchymal transition of mesothelial cells, and angiogenesis. Lastly, we summarize the present and future strategies for developing more biocompatible PD solutions.

## 1. Introduction

All over the world, it is estimated that 2 million people suffer from end-stage renal disease (ESRD), and this number continues to increase every year, representing an important economic problem [1]. The ideal treatment for ESRD would be kidney transplantation, but, in the absence of this availability, most patients undergo dialysis. Peritoneal dialysis (PD) is a well-established renal replacement treatment that several studies have shown to be safe and as efficacious as hemodialysis [2,3,4].

With respect to hemodialysis, PD has a series of advantages: it is home-based and thus cost-saving [5], allowing a superior quality of life, it preserves better the residual renal function, while at the same time it produces a gradual and continuous solute and fluid exchange with minimal cardiac stress [6,7,8].

Although PD has a strong potential, the proportion of ESRD patients treated with this technique in developed countries is consistently lower compared to hemodialysis (about 13% in Europe and 10% in the USA) [6,9,10], well below the optimal estimated utilization rate of 25–30% [11]. On the whole, the sub-optimal utilization of PD might be due to financial and economic reasons that favor hemodialysis, lack of patient information about this renal replacement therapy option, or fear of complications and side effects [12]. Another reason is the concern about the durability of the technique as it may be limited by peritoneal membrane integrity and capacity to sustain the treatment over time. It has been proved that peritoneal membrane dysfunction is responsible for about 30% of technique failure [13], and clinical studies showed that peritoneal ultrafiltration (UF) gradually declines 2–4 years after the initiation of PD [14,15]. In the short and medium period, the main causes of PD failure are infections (mainly peritonitis) and issues with the catheter [16,17], whereas, in the long period, the principal problem is the bio-incompatibility of PD solutions which do not preserve the integrity and functionality of peritoneal membrane [18]. Consequently, novel strategies to slow peritoneal membrane deterioration are desirable to allow a significant diffusion of PD, considering its higher economic and environmental sustainability than HD [6].

## 2. PD Technique

In PD, the peritoneum, the membrane covering the entire peritoneal cavity, is used as a dialysis membrane because it is highly vascularized ad has a large surface area. The parietal peritoneum comprises a single layer of mesothelial cells and a sub-mesothelial area. The mesothelial cells line the peritoneal cavity. Below, the sub-mesothelial zone contains the interstitium, which is a gel-like matrix containing fibroblasts, mast cells, collagen, and other extracellular matrix material. The third layer contains a network of capillary endothelium, endothelial basement membrane, and a capillary fluid film overlying the endothelium [19,20].

PD removes excess water by osmosis and electrolytes as well as metabolic waste products by diffusion across a concentration gradient between the capillary blood and the PD fluid infused into the peritoneal cavity via an implanted intra-abdominal catheter. Usually, two liters of PD solutions are infused into the peritoneal cavity and the effluent is drained after some hours (4 to 8 h are typical dwell times). This procedure is then repeated manually about four times daily (continuous ambulatory PD; CAPD) or using a cycler during the night (automated PD; APD). The solute and water transport across the peritoneal membrane is explained via the three-pore model [21]. In brief, solute and water transport occurs across the vascular endothelium through three pores of varying sizes: large, small, and ultra-small. The large pores (1%) are formed by inter-endothelial cell gaps and are the main site for macromolecule and protein transport. Moreover, the small pores are formed by inter-endothelial gaps, but they account for over 95% of both solutes and water solute removal. Ultra-small pores, made up of aquaporin-1, have been described in endothelial cells of the peritoneal membrane and function as transcellular channels that, under the influence of an osmotic gradient, allow the movement of water only [21,22].

PD solution generally contains the physiological amount of electrolytes (chloride, calcium, sodium, and magnesium), a buffer to correct uremic acidosis (bicarbonate and lactate), and an osmotic agent to induce peritoneal ultrafiltration (UF). Glucose is the main used osmotic agent because it is highly effective, has a low cost, and has a satisfactory safety profile. However, to create an osmotic gradient for the removal of electrolytes and toxins in convection with water, glucose is used 10- to 50-folds higher than serum concentration and this constitutes the principal issue of PD solution bio-incompatibility.

PD removes excess water by osmosis and electrolytes as well as metabolic waste products by diffusion across a concentration gradient between the capillary blood and the PD solution infused into the peritoneal cavity via an implanted intra-abdominal catheter. PD solution contains physiological amounts of electrolytes, a buffer to correct uremic acidosis (bicarbonate or lactate), and an osmotic agent to induce peritoneal UF. Glucose is the main used osmotic agent because it is highly effective, has a low cost, and has a satisfactory safety profile. However, to create an effective osmotic gradient, glucose is used 10- to 50- folds higher than serum concentration and this constitutes the principal issue of PD solution bio-incompatibility.

A multiplicity of studies has proved that long-term exposure to the peritoneum with an un-physiological PD solution activates a series of pathological events such as changes in peritoneal vasculature solutes (neoangiogenesis) with a consequently increased transport of small changes in the interstitium (fibrosis) with a consequent reduction of osmotic conductance as well as the recruitment of inflammatory cells and increased production of inflammatory cytokines which worsen and fuel the un-physiological situation [13,23]. Consequently, the consequent loss of peritoneal integrity reduces UF capacity and PD drop-out [24].

## 3. Pathophysiology of Peritoneal Membrane Failure (Fibrosis, EMT, Angiogenesis)

The unphysiological characteristics of PD solutions and the uremic status are considered as main factors leading to the functional decline of the peritoneal membrane [25]. These factors induce a chronic peritoneal inflammation that can be worsened by episodes of peritonitis. Structural changes of the peritoneal membrane, including loss of mesothelial cells monolayer, sub-mesothelial fibrosis, angiogenesis, and hyalinizing vasculopathy, are the consequence of reparative processes to inflammatory insults [26,27].

Indeed, inflammation induces neoangiogenesis that increases the surface area available for solute diffusion, and on the other hand, fibrotic thickening of the peritoneum increases flux resistance and consequently reduces water flow. Therefore, initial UF decline is related to increased solute transport and consequent dissipation of the osmotic gradient. Moreover, the onset of fibrosis and neovascularization contribute to increased small-solute transport and UF failure [28].

Peritoneal fibrosis is a slow process, but functional alterations are detectable well before structural changes [29]; moreover, some authors reported that sub-mesothelial thickening and vascular changes could be present even without signs of mesothelial cell layer loss [26].

The first features of peritoneal fibrosis in PD patients were described in the 1980s [28] and subsequently, it has been proved that uremic condition and PD duration are responsible for the development of peritoneal deterioration [26,30]. Macroscopically, the peritoneum exposed to dialysate is brownish or tanned, and it displays texture alterations such as the loss of surface moisture [31]. Histologically, the first alterations occur in the mesothelial layer with distinctive cytoplasmic inclusion and signs of focal defoliation [32]. The subsequent alteration involves the sub-mesothelial compartment. Importantly, sub-mesothelial thickness and vascular alteration are associated with the duration of PD and UF failure [26].

Over the last twenty years, it has been proved that fibrosis, inflammation, angiogenesis, and epithelial-to-mesenchymal transition (EMT) are tightly interconnected in the pathogenesis of UF failure [23,33]. EMT is a common process during physiological situations such as development and wound healing but also in pathological events such as cancer and organ fibrosis [34]. In the peritoneum, the correct definition of EMT is a mesothelial-to-mesenchymal transition (MMT).

MMT represents a complex phenomenon of cellular trans-differentiation that converts the mesothelial phenotype into a mesenchymal one, with the loss of epithelial characteristics and the acquisition of mesenchymal features [35]. MMT was initially thought of as irreversible, but several studies have shown that it is potentially reversible [36]. During MMT, mesothelial cells lose cell polarization, undergo the disassembly of cellular contacts such as adherent and tight junctions, and, at the same time, acquire a fibroblastic shape characterized by higher motility and the capacity to produce and secrete extracellular matrix (ECM). Given these characteristics, mesothelial cells that underwent MMT can migrate to the sub-mesothelial zone and secrete ECM, thus contributing to fibrosis [35]. However, there is still an ongoing debate about the individual contribution of MMT-derived fibroblasts to the pool of sub-mesothelial activated fibroblasts with respect to the activated resident stromal fibroblasts [23]. Moreover, also endothelial-to-mesenchymal transition (EndoMT) could contribute to the pool of activated sub-mesothelial fibroblasts [37], as it occurs in the onset of fibrosis in other districts [38].

The earliest event in MMT involves the loss of cell-to-cell contact, which is associated with the downregulation of epithelial markers such as E-cadherin, cytokeratin, and zonula-occludens-1 (ZO-1) [39,40]. Tight junction proteins such as claudins and occludins are para-cellular components which regulate the transport in the peritoneal mesothelium and their expression and localization are altered in PD patients [41]. In addition, loss of mesothelial layer integrity induces sub-mesothelial tissue to come into contact with bio-incompatible PD solutions as well as inflammatory cytokines [42]. However, it must be kept in mind that as mesothelial cells are of mesodermal origin, they co-express in basal conditions with both epithelial and mesenchymal markers. This may explicate their higher plasticity. Regarding epithelial markers, these cells express a high amount of epithelial cytokeratins, such as cytokeratin 8–18, and proteins of tight and adherens junctions, such as junctional adhesion molecule 1 (JAM1) and ZO-1. E-cadherin is expressed on the membrane and cytoplasm of mesothelial cells [43]. Like mesenchymal cells, mesothelial cells express the intermediate filaments vimentin and desmin constitutively [35,44]. E-cadherin downregulation is due to the induction of Snail, a master factor of EMT, directly inhibiting the E-cadherin transcription [35].

Other possible causes of bio-incompatibility of PD solutions are hypertonicity for the generation of crystalloid osmosis [45], glucose degradation products (GDPs) formed during heat sterilization [46,47], as well as advanced glycation end products (AGEs) formed in the peritoneal cavity [48,49].

UF failure is associated with the increased vascular surface area due to neo-angiogenesis. Vascular wall thickening and augmented permeability increase small solute permeability [50,51,52]. Experimental studies proved increased VEGF production was associated with the use of standard PD solutions and the time of dialysis vintage [53]. Interestingly, VEGF levels decreased when patients were switched from a glucose-based to a glucose-free PD solution (icodextrin, glycerol, and amino acid-based dialysis solutions), suggesting a central role of high glucose concentration in the upregulation of peritoneal VEGF production [54].

The connection between angiogenesis and EMT is well recognized. EMT in mesothelial cells is associated with increased levels of peritoneal VEGF [55,56,57]. Expression of VEGF is firmly controlled at several steps: transcription, mRNA stabilization, alternative splicing, and translation [58,59]; in addition, different factors and cytokines are usually upregulated during PD (IL-1b, IL-6, IL-17, oxidative stress) can regulate its production [60,61]. In particular, TGF-β, a master supervisor of EMT, increases VEGF expression in mesothelial and fibroblasts cells. Moreover, TGF-β inhibition decreased peritoneal fibrosis and VEGF production in a murine model [62]. VEGF signaling is also regulated by the expression of VEGF receptors and co-receptors [58] and they are modulated in mesothelial cells EMT [61].

## 4. Molecular Pathway of Fibrosis

### 4.1. TGF-Beta/Smad/Non-Smad/Glucose

TGF-β is part of a superfamily that includes different signaling proteins such as bone morphogenic proteins, activins, and TGF-β isoforms [62], which are involved in several physiological and pathological processes, including proliferation, apoptosis, embryonic development, and organ fibrosis [63]. TGF-βsignaling represents a common mediator of peritoneal fibrogenesis induced by glucose, GDPs, and AGEs in bioincompatible PD solutions [64]. Exposure of mesothelial cells to a high glucose dialysate is associated with a higher synthesis of TGF-β [65]. Moreover, TGF-β signaling is amplified after glucose exposure due to the up-regulation of TGF-β receptor types I and II (TGFR1, TGFR2) in mesothelial cells [66]. Protein kinase C-α (PKC-α) is the common signaling pathway driving TGF-β upregulation in mesothelial cells) [67].

GDPs have also been implied in altering mesothelial cell function and proliferation [68], increasing TGF-β expression and extracellular matrix deposition in the peritoneal wall [69]. Clinical studies reported that TGF-β production correlates with PD vintage [70,71], and in-vivo studies prove that exogenous TGF-β overexpression induces peritoneal fibrosis, increases vessel density, and deteriorates solute transport as well as for UF capacity [72,73].

TGF-β1 can transduce signals through Smad-dependent and Smad-independent pathways, even though most profibrotic actions of TGF-b1 run via Smad signaling. In the classical pathway, Smad2/3 are phosphorylated by PKC, activated by TGFR1, and activin receptor I-β (ACTR1B). Subsequently, they are released from the receptor complex to form a heterotrimeric complex with Smad4 and translocate into the nucleus. Here, they regulate the transcription of target genes in collaboration with various coactivators and corepressors [74,75].

Smad7 is a type of inhibitory Smad, which inhibits Smad2/3 phosphorylation by blocking access to TGFRs. Some works highlighted the positive role of Smad7 on peritoneal failures, such as attenuation of PD-induced peritoneal fibrosis, angiogenesis, and inflammation [76,77,78]. On the other side, BMP-7 exerts antagonistic effects on TGF-β as in PD fluid-instilled rats and co-administration of BMP-7 ameliorated peritoneal fibrosis and increased capillary density [79]. Besides, Smad3 inhibition in uremic-PD rat models treated with recombinant BMP7 decreased peritoneal fibrosis, sub-mesothelial capillary density, and increased UF capacity [80,81]. It has also been proved that mesothelial cells constitutively express BMP-7 and that BMP-7-dependent Smads1/5/8 are reduced in response to conventional PD solutions [79].

Non-Smad signaling pathways are characterized by the activation of protein kinase C, extracellular signal-regulated kinase (ERK), c-Jun N-terminal kinase (JNK), and phosphatidylinositol-3-kinase activating the serine-/threonine-specific protein kinase [23].

Several data indicate that high glucose mediates the phosphorylation of PKC [67] and MAPK [82]; furthermore, TGF-β exposure up-regulates Akt (also known as protein kinase B), a phosphatidylinositol-3 kinase (PI3K) target indicating the implication of non-Smad signaling in peritoneal EMT and fibrosis [83]. In-vivo models also confirmed that inhibition of JNK and p38 MAPK counteract TGF-β-induced peritoneal fibrosis [84,85,86].

Moreover, NF-κB inhibition has been linked to TGF-β signaling inhibition [87].

Finally, high glucose concentration in PD solutions is tightly connected with TGF-β signaling and UF failure. It has been proposed that the degradation of up-taken glucose induces changes in the intracellular NADH/NAD+ ratio, like hypoxia. Exposure to high levels of glucose stimulates the formation of mediators such as TGF-β and plasminogen activator inhibitor-1. This effect is also associated with a higher expression of glucose transporter 1 (GLUT-1). The increased amount of GLUT1 further enhances intracellular glucose uptake and thereby stimulates the vicious loop, including dialysate glucose exposure, peritoneal fibrosis, and UF failure [88].

### 4.2. Other Signaling Pathway: CTGF, NLRP3/IL-1b, and Cytokines

Connective tissue growth factor (CTGF) is a downstream mediator of TGF-β [89] and induces similar effects: ECM production, cell proliferation, adhesion, and migration [90]. In detail, CTGF expression is activated by TGF-β via a responsive element in the promoter region of the CTGF gene [91] and mediated by Smad3 and Smad4 [92]. Its profibrotic properties have been shown in multiple mesenchymal cells, in which CTGF is a downstream effector of TGF-β [93].

Clinical data demonstrate that CTGF is upregulated in PD patients with UF failure [94,95]; its expression is regulated by glucose [96] and correlates with peritoneal membrane thickness in PD patients with and without EPS [97].

Studies in mouse models proved that also AGEs and GDPs act via CTGF in peritoneal fibrosis, angiogenesis, and inflammation [96,98,99]. Although CTGF is involved in peritoneal fibrosis, additional studies will be necessary to characterize its potential as a pharmacological target as it lacks a specific receptor, it has several isoforms, and it interacts with multiple factors (bone morphogenic factors, VEGF, Wnt, integrins, heparan sulfate proteoglycans, and epidermal growth factor receptor) [100].

Recent data suggest that the NOD-like receptor protein 3 (NLRP3) inflammasome is involved in peritoneal inflammation and consecutive fibrosis. NLRP3 intracellular complex is a component of the innate immune system that mediates caspase-1 activation and regulates the release of pro-inflammatory cytokines IL-1β and IL-18 in response to microbial infection and cellular damage [101]. It has been shown that high glucose-based PD solution activates NLRP3/IL-1β peritoneal mesothelial cells [102,103] and that genetic deficiency of NLRP3 complex or IL-1β reduces inflammatory and peritoneal fibrosis model in mice [104].

Peritoneal injury causes the activation of macrophages, neutrophils, endothelial cells (ECs), and MCs, which are the main sources of proinflammatory cytokines and fibrotic mediators in response to external signals [105,106]. Once activated, they release numerous inflammatory cytokines, including IL-6, IL-1β, IL-8, TNF-α, monocyte chemoattractant protein-1 (MCP-1), and macrophage inflammatory protein 2 [107,108,109].

IL-6 is a crucial actor in modulating peritoneal inflammation. Intraperitoneal IL-6 is associated with increasing peritoneal solute transport rate [110] and intraperitoneal IL-6 production is proportional to dialysate glucose concentration [111]. IL-6 and soluble IL-6 receptors induce the synthesis and secretion of MCP-1, which attracts monocytes and lymphocytes [112]. A recent study proved that IL-6 leads to peritoneal inflammation and fibrosis development via a STAT3-dependent pathway [113]. IL-6 inhibition ameliorated EMT in human peritoneal mesothelial cells in vitro and ameliorated high glucose-mediated peritoneal fibrosis development in vivo via inhibiting STAT3 phosphorylation [113].

Another cytokine involved in the peritoneal inflammation is IL-17, which strongly affects mesothelial cell cytokine production such as CXCL1 [114]. Moreover, IL-17 is present in the peritoneum of PD patients and correlates with both the duration of PD and the extent of peritoneal inflammation and fibrosis [115]. Recent surveys showed that treatment with alanyl-glutamine on rats and mice exposed to PD fluids resulted in a reduction of peritoneal fibrosis associated with reduced peritoneal IL-17 expression [116].

### 4.3. The Role of Metabolism

**Figure 1:**
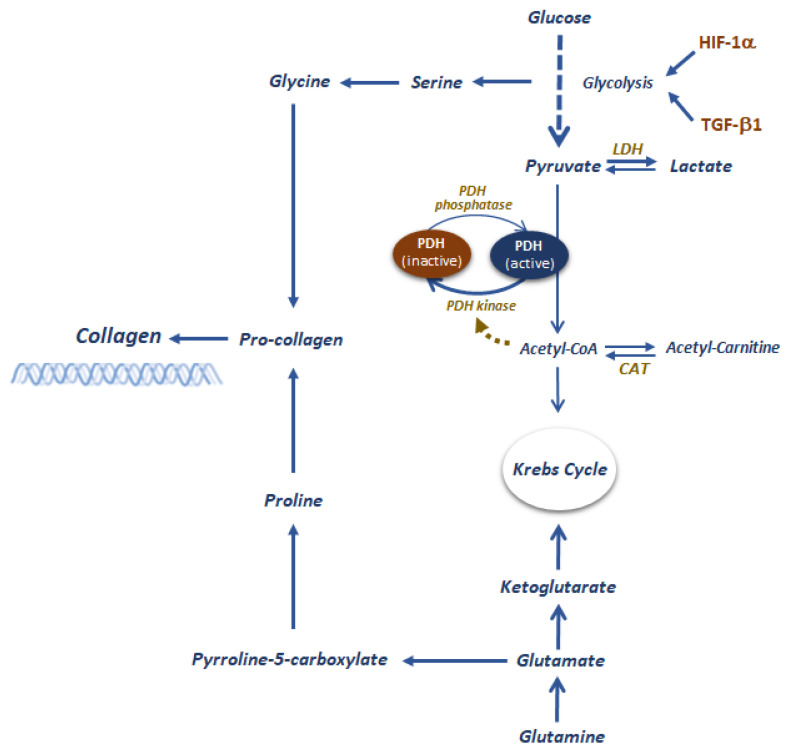
Graphical representation of metabolic control of fibrosis. Several metabolic processes such as glycolysis, glutaminolysis, and fatty acid oxidation contribute to the deposition and other ECM components. High glucose levels activate glycolysis directly by increasing HIF-1a expression, which in turn amplifies the production of TGF-b1. The latter would not only further sustain high glycolytic rates but also fibrogenesis. The increased production of lactate sustains macrophages polarization toward an inflammatory phenotype, which worsens the fibrosis. In addition, the increased glycolytic rate makes glycolytic intermediates available in larger quantities, contributing to the synthesis of amino acid substrates for collagen synthesis. Moreover, glutamine has a role in collagen synthesis, but it can also contribute to ATP production through oxidative phosphorylation. A key metabolic switch in maintaining a chronically activated fibrogenic state is the pyruvate dehydrogenase complex (PDH).

Glycolysis, glutaminolysis, and fatty acid oxidation are metabolic processes that supervise the deposition and breakdown of collagen and other ECM components, which may result in fibrosis [117]. Hyperglycemia upregulates TGF-β and hypoxia-inducible factor 1 subunit alpha (HIF1α) expression [118] by increasing the glycolytic rate and inhibiting pyruvate dehydrogenase complex (PDH), promotes the production of lactic acid [119]. Glycolytic intermediates are important in the synthesis of amino acid substrates for collagen synthesis [120] and lactic acid promotes lactylation of lysine residues in extracellular proteins, favoring the conversion of macrophages to an inflammatory phenotype [121]. In summary, hyperglycemia increases TGF-β and HIF1α expression, which in turn elevates rates of glycolysis and lactic acid production with the consequently increased collagen synthesis, acidification, expansion, and lower degradation of ECM. In essence, a pathway promoting and sustaining fibrosis. The substrate glutamine also has a role in EMT. This amino acid is important for collagen synthesis, but it can be converted to glutamate and then to ketoglutarate, which provides a substrate for the generation of NADH and FADH2 and consequently ATP through oxidative phosphorylation [122,123,124] (Figure 1).

## 5. Effluent Biomarkers to Monitor PD Efficiency

Prognostic biomarkers have been proposed in PD patients to evaluate the peritoneal membrane deterioration. The ideal PD biomarker could be directly accessible in PD effluents to allow one to identify PD patients who are at a high risk of complications. The main biomarkers currently used in PD are IL-6, a marker of chronic peritoneal inflammation, and cancer antigen-125 (CA-125), which is an expression of mesothelial cell mass [125].

IL-6 increases in the effluent of patients with acute bacterial peritonitis and may be used to evaluate the bacterial clearance during the infection. Furthermore, IL-6 in PD effluent correlates with subclinical infections (e.g., biofilms on PD catheter) [126]. Notably, experimental studies suggest persistent peritoneal IL-6 is associated with membrane change/fibrosis and angiogenesis. Other interleukins (IL-8, IL-17) are investigated to evaluate a potential role as an inflammatory marker [125].

The peritoneal membrane undergoes progressive remodeling over PD time resulting in the accumulation of extracellular matrix and fibrosis, included in a complex process called Peritoneal MMT in which mesothelial cells are transformed into fibroblast-like cells leading to inflammation, fibrosis, and angiogenesis. Peritoneal levels of CA-125 have been proposed to estimate mesothelial cell mass as a surrogate parameter for the peritoneal membrane status. The change over time in CA-125 has been proposed as a marker of MMT though the findings are not conclusive [127].

Micro RNAs M (MiR) are small non-coding RNA molecules (18–24 nucleotides), which work as a post-transcriptional regulator in several cellular processes. In PD, microRNA-21 and microRNA-31 were recently proposed to evaluate MMT, but their role is still debated [128].

Recently, PD effluent biomarkers, identified by “omics” technologies, especially proteomics and metabolomics, could predict the onset of peritoneal membrane dysfunction. The metabolic profile in PD effluent might be the expression of a healthy membrane and its change over time predict technique survival [129].

Interestingly, more recently, water channel Aquaporin 1 (AQP1) excreted by the mesothelium has been studied as a biomarker in PD effluent. AQP-1 levels in the effluent correlate with ultrafiltration and free water transport (sodium sieving) evaluated by the peritoneal equilibration test [130].

However, there is no evidence of association of any PD biomarkers with relevant clinical outcomes and their use in the clinical practice is modest.

## 6. Strategies to Reduce Fibrosis

### 6.1. Low GDPs and Neutral pH

New glucose-based solutions with a neutral- or physiological-pH and low-GDP content (using multi-chamber bags) have been developed to increase the biocompatibility of PD dialysate [131]. The use of lactate or bicarbonate as a pH buffer has significantly reduced systemic GDPs and AGEs. However, the clinical superiority of neutral-pH, low-GDP PD solutions has been questioned [132,133]. In detail, neutral-pH, low-GDP PD solutions seem to be better at preserving the peritoneal endothelial glycocalyx compared to conventional acidic solutions during prolonged PD [134]. However, biopsies in children showed early peritoneal inflammation, hypervascularization, fibroblast activation, and epithelial–mesenchymal transition, which affected PD membrane-transport function [135].

### 6.2. Glucose Sparing

The high glucose content of the PD solution is the main culprit for peritoneal damage over time. In addition, exposure to high glucose concentration leads to systemic adverse effects such as hyperglycemia, insulin resistance, diabetes, and cardiovascular diseases [136,137]. Numerous compounds have been tested as alternatives to glucose, but only two osmotic agents are currently available in clinical practice: icodextrin and amino acids. Unfortunately, these compounds can only be used in a single daily peritoneal exchange [138,139], reducing daily glucose load by only 30–50% [140]

Icodextrin is a water-soluble glucose polymer derived from starch. The use of icodextrin-containing PD solution is associated with improved peritoneal UF and fewer episodes of fluid overload [141]. However, the low pH of the icodextrin solution may induce an increased local and systemic inflammation and activation of the EMT process [142].

PD solutions containing amino acids (e.g., Nutrineal^®^) have a pH of 6.7 and are free of GDPs. This PD solution may improve the nutritional status of some malnourished PD patients by increasing muscle amino acid uptake [143]. Peritoneal ultrafiltration rate and small solute clearance over a 6-h dwell did not show any main difference between amino acid-based PD fluid and equimolar glucose-based solutions [144,145]. However, the biocompatibility of these PD solutions, which influences the peritoneal function over time, is debated. Indeed, while some experimental studies showed a better biocompatible profile compared to standard glucose-based PD solutions, others reported increased generation of nitric oxide in human mesothelial cells cultured with a PD solution containing amino acids, a finding that may have pathophysiological relevance [146].

Other compounds that have been tested for a potential use in the PD solution as osmotic agents to replace glucose include taurine and hyperbranched polyglycerol, but they are under experimental development.

### 6.3. Use of Metabolically Active Osmolytes

The osmo-metabolic approach uses osmolytes in the PD solution, which may offer a bioactive glucose-sparing by reducing intraperitoneal glucose load without compromising UF and mitigating the underlying systemic negative metabolic effects caused by the glucose load.

L-carnitine (LC) and xylitol may be used as osmo-metabolic agents in PD dialysate. LC is a naturally occurring compound involved in fatty acid oxidation [147]. The mode of action of LC relates to its ability to modulate intra-mitochondrial acetyl-CoA levels, a key metabolic intermediate able to affect both muscle glucose disposal and liver glucose production [148]. Xylitol, a five-carbon sugar alcohol, is a physiologic metabolic intermediate of the glucuronate-xylulose cycle, a pathway very active in the liver and intimately interconnected with the pentose monophosphate shunt at the level of D-xylulose-5-phosphate [149,150]. Interestingly, xylitol is a very poor insulin secretagogue compared to glucose [151]. A key attribute of xylitol is that it does not undergo a Maillard reaction as it usually happens between traditional reducing sugars (i.e., glucose) and amino acids/proteins, a reaction also commonly responsible for the formation of AGEs [152].

LC and xylitol are characterized by molecular weight similar to glucose, high water solubility, and osmotic properties [148]. The good biocompatibility of LC- and xylitol-containing solutions has been demonstrated in several in-vitro and in-vivo models [153,154] In addition, clinical studies have demonstrated excellent tolerability and feasibility of xylitol [155] and L-carnitine solutions [156], as well as a better preservation of urine volume, compared to controls (treated with standard glucose-based PD fluids) over a 4-month period [157]. Clinical use of xylitol- or LC-containing dialysate in CAPD patients was associated with positive metabolic effects such as improving glycemic control.

PD solution containing LC, xylitol, and low glucose, has been designed to achieve a favorable synergistic combination of the two osmo-metabolic agents. In-vitro studies provide further evidence that this novel formulation of PD solutions better preserves the integrity of the mesothelial cell layer compared to conventional PD solutions reducing fibrogenic features and inflammation [158,159].

Preliminary results obtained from a phase II, prospective, open, multicenter study to investigate the tolerability and the efficacy of osmo-metabolic agent-based PD solutions in CAPD patients (NCT04001036) confirmed that these novel solutions are well tolerated and no serious adverse reactions were reported. Non-inferiority of the osmo-metabolic agent-based PD solutions compared to standard solutions in terms of peritoneal transport and adequacy also was demonstrated as targets [160].

### 6.4. Use of Pharmacological Agents Added to Conventional PD Solutions

To counteract the adverse effects of conventional PD solutions, several compounds have been tested in-vitro and in-vivo. Unfractionated heparin, low-molecular-weight heparins, and sulodexide showed a different response in clinical trials, probably due to their different capacity to inhibit complement [161,162,163,164]. With the same objective to inhibit complement, sodium citrate has been tested in association with heparin [165].

A recently tested strategy is the addition of pharmacological doses of alanyl-glutamine (Ala-Gln) to glucose-based PD solutions and phase II clinical trials indicate that Ala-Gln supplementation in PD solution improves biomarkers of peritoneal membrane integrity, immune competence, and systemic inflammation when compared to non-supplemented PD solution with neutral pH and low-glucose degradation probably via an antioxidant mechanism [166,167,168]. However, use in clinical practice remains still debated.

Another element that has been tested as a possible pharmacological agent to add to the DP solution is molecular hydrogen (H2) [169]. Its antioxidant and anti-inflammatory properties have been tested in various animal models [170]. Molecular hydrogen, added to a standard PD solution, has also been tested in humans (6 patients), confirming a reduction in oxidative stress at both the peritoneal and systemic levels in the absence of adverse events [171]. In addition, recent in-vivo studies indicate that molecular hydrogen could preserve mesothelial integrity and reduce the progression of glucose-induced fibrosis [172,173]; thus, future clinical studies will be necessary to evaluate the efficacy and safety of this therapeutic solution.

Finally, a recent study proposes the addition of lithium chloride to conventional PD solutions for preserving peritoneal membrane integrity [174]. In detail, lithium chloride could reduce apoptosis, peritoneal membrane fibrosis, and angiogenesis by regulating the activity of some kinases, such as glycogen synthase kinase 3 and protein kinase 2. Although these findings may be hopeful, the real benefits will have to be demonstrated, considering that lithium chloride is an antidepressant and potentially nephrotoxic agent.

Even if the addition of pharmacological agents can improve the characteristics of traditional dialysis solutions, the glucose concentration remains very high, and consequently, the harmful effects associated with it would remain present [132,146]

### 6.5. Glycolytic and Pyruvate Metabolism as Targets to Control Peritoneal Fibrosis

**Figure 2:**
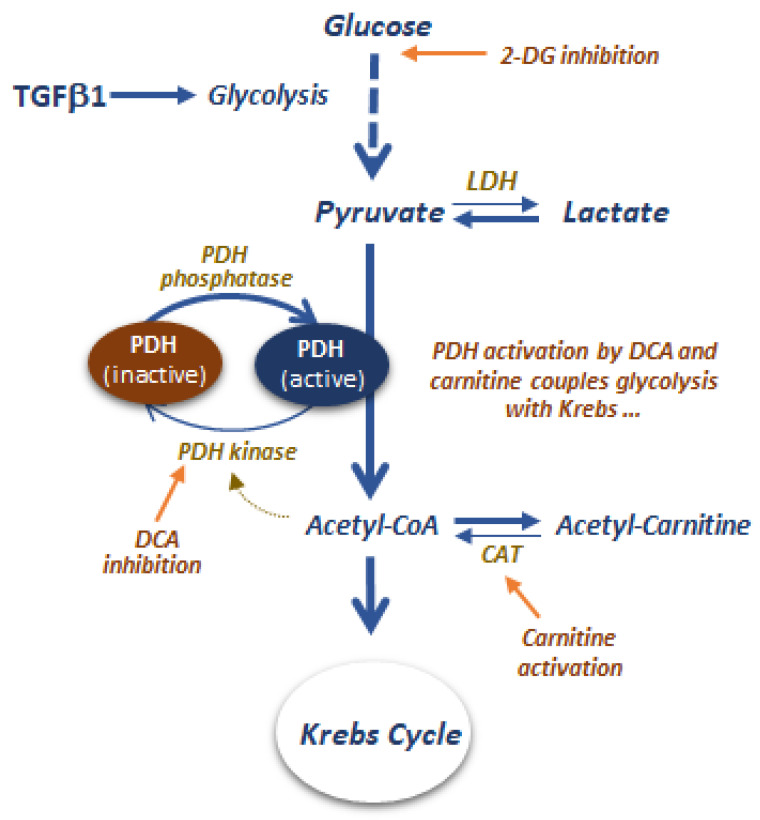
Metabolic strategies to control fibrosis could be based on inhibiting glycolysis, fatty acid, and pyruvate oxidation. Glycolysis can be modulated by inhibiting hexokinase 2 by 2-deoxyglucose, though the safety of this approach must be proved. The alternative strategy is coupling glycolysis with the Krebs cycle by inhibiting PDH Kinase using DCA or increasing PDH activity by reducing the intramitochondrial acetyl-CoA pool using L-carnitine.

An alternative pharmacological strategy to control fibrosis could be based on a fatty acid or pyruvate oxidation or even by inhibiting glycolysis. For instance, an increase of PDH, a key enzyme in coupling glycolysis with the Krebs cycle, can be achieved through the inhibition of pyruvate dehydrogenase activity kinase (PDHK), a potent inhibitor of PDH, using dichloroacetate (DCA) [175]. DCA has been shown to be very effective in inhibiting fibrosis in various experimental models [175,176,177,178,179]. Another means to keep more active PDH is by reducing the intramitochondrial pool of acetyl-CoA, a potent allosteric activator of PDHK, by supraphysiological concentrations of L-carnitine [148]. The latter mechanism involves the freely reversible reaction catalyzed by carnitine acetyltransferase in transferring the acetyl-residue esterified to Coenzyme A to L-carnitine to form acetyl-carnitine. Indeed, as this enzymatic reaction is very sensitive to the mass action effect of L-carnitine, the intramitochondrial concentration of acetyl-CoA will be significantly reduced, translating into a less active PDK1 and, hence, a more active PDH [180]. L-Carnitine administration has been shown to mitigate the induction of fibrosis in various experimental models. A third option may be the inhibiting glycolysis by 2-deoxyglucose, a glucose derivative that acts as an inhibitor of hexokinase 2 and hence of glycolysis [181]. As TGF-β1 is a key facilitator of the EMT transition by switching cellular energy provision from oxidative phosphorylation to substrate-level phosphorylation through aerobic glycolysis [182], the reduction of high glycolytic fluxes with 2-deoxyglucose could reduce peritoneal fibrosis [183,184]. However, according to the mode of action of DCA and L-carnitine, their anti-fibrotic effects may not necessarily require a reduction of glycolytic flux but rather an efficient coupling of such flux with an active PDH. In addition, it remains to be established whether the inhibition of glycolysis is a safer strategy compared to the diversion of pyruvate metabolism towards oxidative phosphorylation [185] (Figure 2).
